# A comparative analysis of short-term results in range of motion following arthroscopic arthrolysis with vs. without peripheral nerve block in cases of elbow stiffness

**DOI:** 10.1016/j.jseint.2024.10.009

**Published:** 2024-11-16

**Authors:** Tamara Babasiz, Michael Hackl, Felix Krane, Lars P. Müller, Tim Leschinger

**Affiliations:** Department of Orthopaedic and Trauma Surgery, Faculty of Medicine and University Hospital Cologne, University of Cologne, Cologne, Germany

**Keywords:** Arthroscopic arthrolysis, Elbow stiffness, Peripheral nerve block, Regional anesthesia, Range of motion, Retrospective study

## Abstract

**Background:**

This study aimed to investigate the clinical short-term results in patients with elbow stiffness, particularly focusing on the range of motion (ROM) following arthroscopic arthrolysis. Our objective was to assess potential differences in postoperative outcomes between patients who received an additional peripheral nerve block with postoperative nerve block catheter, and those who exclusively underwent general anesthesia at 6 weeks, 3 months, and 6 months after surgery.

**Methods:**

A single-center study was performed on patients undergoing arthroscopic elbow arthrolysis due to persistent elbow stiffness between 2014 and 2018. The participants were divided into 2 cohorts: One underwent arthroscopic elbow arthrolysis with an additional peripheral nerve block, combined with a postoperative nerve block catheter (group 1), while the other received the procedure without peripheral nerve block (group 2). Standardized assessments of ROM and the Disabilities of the Arm, Shoulder, and Hand score were conducted and analyzed preoperatively and at the 6-week, 3-month, and 6-month follow-up.

**Results:**

A total of 32 patients were included in this study. In group 1 (18 patients), ROM in extension/flexion improved significantly from 95° (±27.17) to 124.4° (±12.7°; *P* = .000012) after 6 months. Similarly, a significant improvement from 150° (±29.1) to 170.6° (±13°; *P* = .0013) was observed after 6 months for ROM in pronation/supination. In contrast, group 2 (14 patients) demonstrated an improvement in elbow motion after 6 months, compared to preoperative values, although this increase did not reach statistical significance after 6 months (ROM extension/flexion, *P* = .6016; ROM pronation/supination, *P* = .2461). Furthermore, a significant difference (*P* = .0199) in the delta values of ROM arc for extension/flexion before surgery and after 6 months was identified when comparing both groups, favoring the patient group with additional regional anesthesia (group 1).

**Conclusion:**

Additional peripheral nerve block combined with a postoperative nerve block catheter in arthroscopic arthrolysis in cases of elbow stiffness may be an opportunity to enhance postoperative outcomes by achieving better functional ROM, perhaps through reduced postoperative pain.

Upper extremity function relies heavily on elbow movement for correct positioning of the hand in space. Loss of this movement due to elbow stiffness can cause patients substantial disability.

Elbow stiffness typically arises following elbow injuries.^1^^,^^14^ Approximately 12% of traumatic elbow injuries cause stiffness with significant limitations in movement, which require surgical intervention.^15^ Even a slight reduction in elbow joint mobility can result in significant limitation of functionality, leading to difficulties performing activities of daily life. Sardelli et al demonstrated that a maximum flexion arc of 130° ± 7° and a maximum pronation-supination arc of 103° ± 34° are necessary for sufficient elbow joint function in everyday life.^19^ Furthermore, according to Morrey et al, an elbow joint stiffness is defined as a flexion-extension arc of less than 100° and/or flexion contracture of more than 30°.^14^ These limitations can greatly impact the patient’s overall quality of life. If conservative treatment does not lead to an enhanced range of motion (ROM), fails to attain functional ROM, or patients require a greater level of functionality than achieved, surgical intervention may be considered.^21^ Arthrolysis can be performed either through arthroscopic or open surgery approaches. Primary aims of these procedures are to reduce pain caused by impingement and mechanical issues on the one hand and restore the functional ROM on the other hand.^8^

Studies for shoulder surgery showed that the use of regional nerve blocks effectively reduces the need of patient-controlled analgesia postoperatively.^5^ Active mobilization postsurgery is particularly crucial for optimizing joint mobility outcomes.^1^ This raises the question whether improved postoperative pain control by preoperative regional nerve block, combined with postoperative nerve block catheter during hospitalization, contributes to improved functional ROM outcomes. Thus, the objective of this study was to analyze postoperative results following arthroscopic elbow arthrolysis, specifically focusing on functional ROM at various postoperative time points. The investigation aimed to determine whether patients who underwent general anesthesia with an additional peripheral nerve block catheter exhibited superior postoperative outcomes in functional ROM following arthroscopic arthrolysis compared to those who solely underwent general anesthesia for surgery.

## Methods

A single-center retrospective study was performed on patients, who were treated with elbow arthrolysis between February 17, 2014 and August 14, 2018. We evaluated a total of 43 patients (21 female and 23 male), which were operated during aforementioned period. The indication for surgery, as well as the inclusion criteria, was persistent elbow stiffness with no sufficient improvement after conservative treatment for at least 3 months. Patients who received additional surgical procedures, for example, implant removal or ligament reconstruction were excluded, as were patients who did not complete the postoperative evaluation forms during the specified follow-up periods. The participants were divided into 2 cohorts.

Group 1 included patients, which received arthroscopic arthrolysis under general anesthesia with additional peripheral nerve block, as well as postoperative nerve block catheter during hospitalization. Group 2 included patients, which received arthroscopic arthrolysis only under general anesthesia without additional regional nerve block and postoperative nerve block catheter.

The placement of the peripheral nerve block was performed ultrasound-guided in interscalene position in all cases (group 1). The peripheral nerve block was applicated by the same consultant anesthesiologist, with various attending physicians involved, using 7.5-10 mL 0.5% or 1% Ropivacaine and 7.5-10 mL 1% or 2% Prilocaine, depending on individual patient conditions. Postoperative peripheral nerve block catheters consisted of 0.2% Ropivacaine with a flow of 4-12 mL per hour, based on patients’ pain sensitivity and tolerance, as well as their weight and height.

Patients were evaluated before the surgical procedure for sex; age; dominant limb; need of pain medication; previous operations on the elbow; preoperative condition of the ulnar nerve (McGowan classification);^11^ cause of elbow stiffness and ROM in extension, flexion, pronation, and supination; and disabilities of the arm, shoulder, and hand (DASH) score (Table I).

**Table I:** Demographic characteristics of the 2 groups preoperatively.

	Group 1	Group 2	*P* value
n	18	14	
Age (y)	52.39 (±16.33)	54.64 (±14.49)	.6930
Sex	7 (38.89%) women 11 (61.11%) men	10 (71.43%) women 4 (28.57%) men	.0870
Dominant limb	16/18 (88.89%)	10/14 (71.43%)	.3649
Cause of stiffness			>.9999
Fracture malunion or nonunion	10 (55.56%)	8 (57.14%)	
Osteoarthritis of the joint	4 (22.22%)	3 (21.43%)	
Heterotopic bone formation	2 (11.11%)	1 (7.14%)	
Soft tissue contractures	2 (11.11%)	2 (14.29%)	
Previous elbow operations (≥ 1)	10/18 (55.56%)	7/14 (50%)	>.999
Ulnar nerve state (McGowen)			.3141
Stage 0	11 (61.11%)	9 (64.29%)	
Stage I	7 (38.89%)	4 (28.57%)	
Stage II	0 (0%)	1 (7.14%)	
Stage III	0 (0%)	0 (0%)	
Need of pain medication	7/18 (38.89%)	6/14 (42.86%)	>.9999
ROM (extension/flexion)	95° (±27.17°)	113.93° (±15.21°)	.0348
ROM (pronation/supination)	150° (±29.1°)	161.07° (±40.87°)	.1453
DASH	37.47 (±20.33)	36.97 (±26.09)	>.999

*ROM*, range of motion; *DASH*, disabilities of the arm, shoulder, and hand.

Regarding the cause of stiffness, all patients with primary osteoarthritis of the elbow joint exhibited severe degenerative changes, characterized by significant joint destruction (Broberg and Morrey classification, Grade III).^4^ Patients with elbow stiffness due to heterotopic bone formation demonstrated ectopic bone growth and joint ankylosis, affecting both the pronation-supination and flexion-extension axes (Graham and Hastings classification, Grade IIIC).^6^ Preoperative ulnar nerve condition was categorized using McGowan classification (0: No symptoms; I: Minimal lesions, paresthesia, and dysesthesia, no wasting or weakness of ulnar intrinsic muscles; II: Intermediate lesions, weakness, and wasting of interossei but some voluntary power is retained; III: Severe lesions, paralysis of interossei, and marked weakness of the hand).^11^ Values indicate mean (± standard deviation) or exact values (percentage of whole).

Arthroscopic arthrolysis of the elbow was consistently performed by the same 2 senior elbow surgeons, according to a standardized surgical protocol. The procedure involved the removal of all the impinging bone restricting motion, along with most of the anterior and posterior capsules. The arthroscopy began with accessing the anterior part of the elbow through the anterolateral portal, where the inflow cannula was inserted using a trocar. Subsequently, a high posterolateral portal and a transtricipital portal were established. For the posterior section, the dorsal capsule was carefully detached from its humeral attachment. Additionally, loose bodies were removed, and osteophytes were resected from the olecranon tip, olecranon fossa, and both the lateral and medial regions of the elbow. Attention was then directed to the radiocapitellar joint, where any fibrotic adhesions present were resected. An anteromedial portal was created next, using the inside-out-technique through the pre-existing anterolateral portal. Loose bodies and osteophytes from the coronoid process, coronoid fossa and radial fossa were also removed. Finally, an anterior capsulotomy was performed to enhance elbow extension. Before closing up, passive mobilization was conducted to assess the final ROM. The skin portals were then sutured, and no drains were placed.^13^^,^^18^ No intervention on the ulnar nerve was performed. While the specific course of the surgery may vary depending on intraoperative findings and individual patient conditions, the fundamental standardized procedure remained consistent across all cases.

After surgical intervention, all patients underwent a standardized clinical examination, which included the assessment of clinical outcomes using the measurement of ROM (extension, flexion, pronation, and supination), the DASH score at 6 weeks, 3 months, and 6 months after surgery. Additionally, the question whether patients would choose to undergo arthroscopic arthrolysis once more was examined. ROM was measured using a hand-held universal goniometer, with careful positioning of the upper extremity. For flexion-extension measurements, the forearm was placed in full supination when possible, ensuring the elbow axis (the line connecting the epicondyles) remained parallel to the floor. For pronation-supination measurements, the elbow was bent at 90°, with the arm positioned alongside the chest, and forearm rotation was assessed using the extended thumb. Measurements of ROM were assessed by one physician who served as an assistant during the surgical procedures.

### Postoperative management

The postoperative rehabilitation protocol was consistent for all patients. Exercises were conducted under the guidance of a clinical physician, following a standardized physical therapy protocol. Immediately postoperatively, active assisted functional exercises for the operated elbow were initiated and supervised by our inpatient physiotherapy team. Progressive active motion of the elbow was permitted 1 day after surgery. Elbow protection was advised for 6 weeks, avoiding heavy lifting, use of force against resistance by the affected arm, and monotonous activities of the fingers. All patients received postoperative pain management according to the clinical standard World Health Organization step III protocol, which includes nonsteroidal anti-inflammatory drugs combined with high-potency opioids. Follow-up at our outpatient clinic took place at 6 weeks, 3 months, and 6 months postsurgery and consisted particularly of physical examination, which encompassed, among other assessments, the measurements of ROM and DASH scores. Aside from the fact that patients in group 1 underwent the placement of a peripheral nerve block catheter throughout the postoperative period, postoperative management was performed identically in both groups.

### Statistical analysis

Statistical analysis was performed using GraphPad Prism 10 (GraphPad, San Diego, CA, USA). To compare 2 groups, an unpaired Student’s *t*-test was used for normally distributed data (age). Chi-square with Fisher’s exact test was used for categorical variables (sex, dominant limb, cause of stiffness, previous operations, need of pain medication, and ulnar nerve condition). Distribution of the clinical outcome parameters was presented by the mean, standard deviation, and minimum and maximum values. The normality assumption was checked visually and by using the Kolmogorov-Smirnov test. Hereby, no normal distribution could be assumed for ROM and DASH values. Two-way analysis of variance was used to analyze data in both groups, as well as Mann-Whitney U test to analyze differences between both groups. To calculate the Minimal Clinically Important Difference (MCID), we employed the most commonly used distribution-based method, defined as MCID = 0.5 × standard deviation of the Δ.^22^ The alpha level was set to 0.05.

## Results

A total of 43 patients (21 female and 23 male) treated with elbow arthrolysis due to persistent elbow stiffness were recruited for this study. The causes of elbow stiffness were categorized as primary osteoarthritis of the joint (Broberg and Morrey classification),^4^ post-traumatic osteoarthritis resulting from fracture malunion or nonunion, soft tissue contractures, or the presence of heterotopic bone formation (Graham and Hastings classification)^6^ (Table I). Upon application of the specified criteria, the study cohort comprised 32 patients. The participants were divided into 2 cohorts. Group 1 consisted of 7 women and 11 men, with a mean age of 52.4 years (range: 27-84 years). Group 2 consisted of 10 women and 4 men, with a mean age of 54.6 years (range: 24-80 years) (patient characteristics presented in Table I). The placement of a peripheral nerve block catheter throughout the postoperative period in patients of group 1 extended to an average duration of 3.17 (±1.3) days. No surgical complications or catheter-associated complications were observed in either group.

In group 1, preoperative ROM arc for extension/flexion improved significantly from 95° (±27.17) to 112.8° (±18.4°; *P* = .0335) after 6 weeks, to 118.9° (±15.6°; *P* = .0008) after 3 months, and to 124.4° (±12.7°; *P* = .000012) after 6 months. Preoperative ROM arc for pronation/supination improved from 150° (±29.1) to 161.1° (±18.4°; *P* = .0768) after 6 weeks, to 161.7° (±19.2°; *P* = .0634) after 3 months, and increased significantly to 170.6° (±13°; *P* = .0013) after 6 months.

**Figure 1:**
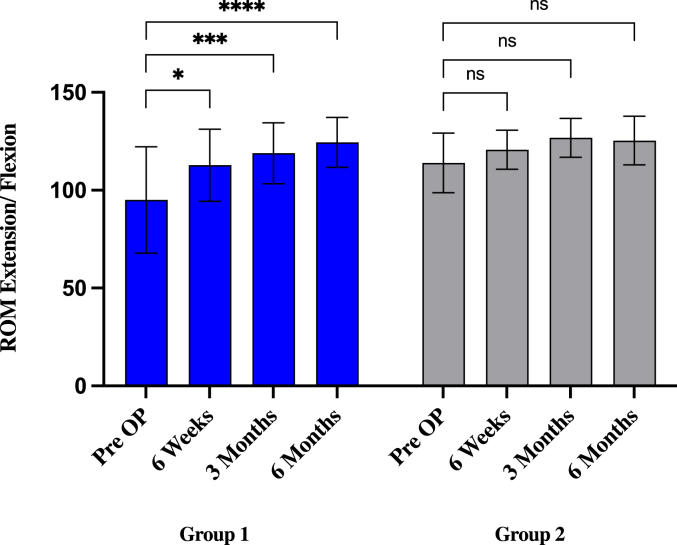
ROM extension/flexion values compared to preoperative status and multiple postoperative time point (6 weeks, 3 months, and 6 months) in groups 1 and 2. ∗*P* < .05; ∗∗*P* < .01; ∗∗∗*P* = .001; ∗∗∗∗*P* = .0001, ns = nonsignificant (*P* > .05). Statistics were performed using 2-way ANOVA test. *ROM*, range of motion; *ANOVA*, analysis of variance.

**Figure 2:**
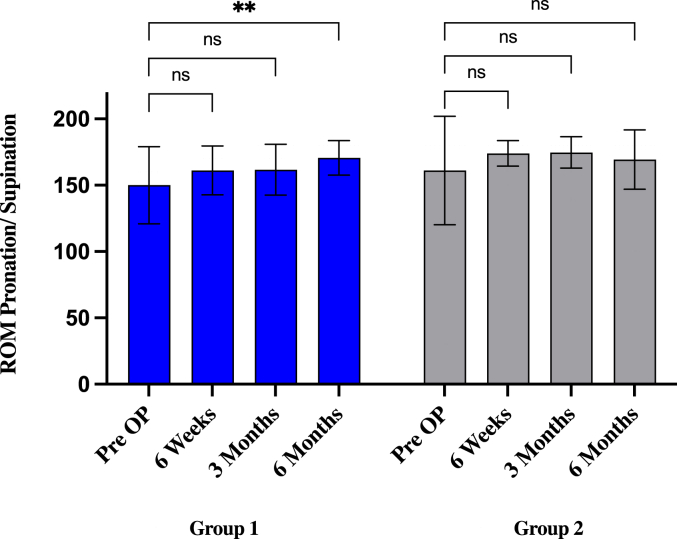
ROM pronation/supination values compared to preoperative status and multiple postoperative time point (6 weeks, 3 months, and 6 months) in groups 1 and 2. ∗*P* < .05; ∗∗*P* < .01; ∗∗∗*P* = .001; ∗∗∗∗*P* = .0001, ns = nonsignificant (*P* > .05). Statistics were performed using 2-way ANOVA test. *ROM*, range of motion; *ANOVA*, analysis of variance.

In group 2, preoperative ROM arc for extension/flexion improved from 113.9° (±15.2) to 120.7° (±9.9°; *P* = .9586) after 6 weeks and increased to 126.8° (±9.9°; *P* = .4481) after 3 months and decreased 125.4° (±12.5°; *P* = .6016) after 6 months. The improvement showed no statistical significance. Preoperative ROM arc for pronation/supination improved not significantly from 161.1° (±40.9) to 173.9° (±9.6°; *P* = .071) after 6 weeks and to 174.6° (±11.8°; *P* = .057) after 3 months and decreased to 169.3° (±22.3°; *P* = .2461) after 6 months (Fig. 1, Fig. 2, Supplemental Table S1).

When comparing the delta values of ROM arc for extension/flexion preoperatively, after 6 weeks (*P* = .2456) and after 3 months (*P* = .2009) between group 1 and group 2, there was no significant difference. Comparing the delta values of ROM arc for extension/flexion before surgery and after 6 months between both groups, we found a significant difference (*P* = .0199). When comparing the delta values of ROM arc for pronation/supination preoperatively and after 6 weeks (*P* = .8668), after 3 months (*P* = .6669), and after 6 months (*P* = .2736) between group 1 and group 2, there was no significant difference (Table II). To evaluate the clinical relevance of our findings, we calculated the MCID. Here, MCID values were calculated for the different postoperative time points after 6 weeks, 3 months, and 6 months (6 weeks: ROM Ex/Flex: 11.2°, ROM Pro/Sup: 16.74°; 3 months: ROM Ex/Flex: 10.95°, ROM Pro/Sup: 17.47°; 6 months: ROM Ex/Flex: 11.3°, ROM Pro/Sup: 14.48°). Importantly, ROM values of group 1 exceeded the MCID at all postoperative time points (except for ROM Pro/Sup at 6 weeks and 3 months), indicating a consistent clinically significant improvement. In contrast, group 2 only had a delta value for ROM Ex/Flex (11.43°) at 6 months that briefly approached the calculated MCID of 11.3° (Table II).

**Table II:** Differences of the delta Δ-values of preoperative ROM and ROM after different postoperative time points between group 1 and group 2.

	Group 1 (G1)	Δ-value	Group 2 (G2)	Δ-value	G1/G2 *P* value
Preoperative					
ROM Ex/Flex	95° (±27.17°)		113.93° (±15.21°)		
ROM Pro/Sup	150° (±29.1°)		161.07° (±40.87°)		
6 weeks					
ROM Ex/Flex	112.8° (±18.4°)	17.78 (±23.9°)	120.7° (±9.9°)	6.79 (±19.38°)	.2456
ROM Pro/Sup	161.1° (±18.4°)	11.11 (±29.48°)	173.9° (±9.6°)	12.86 (±39.16°)	.8668
3 mo					
ROM Ex/Flex	118.9° (±15.6°)	23.89 (±22.85°)	126.8° (±9.9°)	12.86 (±19.68°)	.2009
ROM Pro/Sup	161.7° (±19.2°)	11.67 (±37.46°)	174.6° (±11.8°)	13.57 (±32.78°)	.6669
6 mo					
ROM Ex/Flex	124.4° (±12.7°)	29.44 (±22.16°)	125.4° (±12.5°)	11.43 (±19.46°)	.0199
ROM Pro/Sup	170.6° (±13°)	20.56 (±33.69°)	169.3° (±22.3°)	8.21 (±20.53°)	.2736

*ROM*, range of motion; *Ex/Flex*, Extension, Flexion; *Pro/Sup*, Pronation/Supination.

Delta Δ-values of preoperative ROM; values and ROM; values after 6 weeks, 3 mo, and 6 mo postoperatively; and *P* values between Δ-values (G1/G2 *P* values) are presented. Values indicate mean (± standard deviation) or exact values.

In group 1, the mean DASH score was 37.47 points (±20.33 points) preoperatively, improving to 27.44 points (±20.22 points, *P* = .9952) after 6 weeks, 16.24 points (±16.22 points; *P* = .1175) after 3 months, and 11.55 points (±7.71 points, *P* = .0159) after 6 months.

In group 2, the mean DASH score before surgical intervention was 36.97 points (±26.09 points). The DASH score improved after 6 weeks with a score of 34.99 points (±26.02 points, *P* = .9999), 28.31 points (±28.77 points, *P* = .9999) after 3 months, and 20.39 points (±21.85 points, *P* = .749) after 6 months.

After 6 months, all patients in group 1 (100%) and 12 patients in group 2 (85.71%) would decide for the respective surgery again.

## Discussion

The present study investigated the impact of an additional peripheral nerve block, combined with a postoperative nerve block catheter during hospitalization on short-term postoperative elbow motion following arthroscopic arthrolysis. Effective postoperative pain management is necessary for achieving early ROM and enhancing rehabilitation compliance.^9^ A meta-analysis focusing on postoperative pain management for shoulder arthroscopy previously underscored the high efficacy of peripheral nerve blocks in reducing postoperative pain.^10^ Furthermore, several studies have reported the effectiveness of continuous nerve blocks in postoperative pain management.^3^^,^^12^ Especially, active mobilization after surgery is considered essential for optimizing joint mobility outcomes.^1^ We addressed the question whether a regional nerve block before arthroscopic elbow arthrolysis, coupled with a postoperative nerve block catheter, could enhance the postoperative results for patients with elbow stiffness, specifically concerning elbow motion. We hypothesized that the administration of a regional nerve block before surgery, combined with a postoperative nerve block catheter, may facilitate improved postoperative mobilization by reducing early postoperative pain which might have a significant influence on functional outcome.

Our findings reveal a significant enhancement in elbow ROM in patients who received an additional regional nerve block, coupled with a postoperative nerve block catheter, during the whole follow-up period. Conversely, those who underwent arthroscopic arthrolysis under general anesthesia without the supplemental nerve block also experienced an increase in elbow motion. However, this improvement did not reach statistical significance when compared to preoperative values. Additionally, we found a significant difference between both groups, when comparing the delta values of ROM arc for extension/flexion before surgery and after 6 months. Overall, our results do support our hypothesis that the implementation of a peripheral nerve block, in conjunction with a postoperative nerve block catheter, contributes to enhanced elbow motion following arthroscopic arthrolysis, in contrast to patients who underwent solely general anesthesia.

Interestingly, there is a notable gap in prior research, as no studies have specifically explored the impact of a peripheral nerve block on postoperative elbow ROM following arthroscopic arthrolysis for a stiff elbow. Overall, a few prior studies on postinterventional gain in joint motion for the shoulder exist. Haque et al demonstrated that suprascapular nerve block increased patients’ pain tolerability for joint mobilization, when investigating the treatment of adhesive capsulitis of the shoulder. Interestingly, this effect was persistent even at 3 months following the injection.^5^ Thus, it seems likely that regional nerve block indeed may lead to improved pain-free mobilization even after several months. This aligns with our study’s data on elbow joint mobility, where a substantial improvement in elbow motion was observed 6 months postoperatively, reflecting the initial advantages in postoperative mobilization. Moreover, in another study examining conservative treatment for shoulder stiffness using suprascapular nerve block to restore shoulder motion, a notable reduction in pain severity and disability was observed within 10 days after the block.^17^ This further supports the hypothesis that regional nerve block leads to less pain and better postinterventional joint mobilization. In the exploration of arthroscopic capsular release of the shoulder, the analyzed benefits of a short hospital stay include mainly improved postoperative pain control and early physical therapy by initiation of continuous passive motion.^16^^,^^23^ Ideally, early motion after arthroscopic capsular release of the shoulder should begin within 24 hours to minimize scar tissue formation.^9^ Overall, facilitating early mobilization may be attainable by providing immediate postoperative pain relief through preoperative regional nerve block and postoperative nerve block catheter. Our findings suggest that in the context of arthroscopic elbow arthrolysis, the utilization of a regional nerve block, combined with a postoperative nerve block catheter, significantly contributes to improved postoperative outcomes in terms of ROM, a benefit that may remain discernible for up to 6 months.

According to Steinmann and Adams, the use of regional anesthesia in elbow arthroscopy also introduces some risks. Due to regional anesthesia, a substantial nerve injury may not be apparent and potentially cannot be noticed in the initial postoperative days, while it remains unclear whether regional anesthesia technique may have induced the nerve dysfunction.^2^^,^^20^ However, recent extensive case series indicate neurological complications for arthroscopic elbow arthrolysis at approximately 2%.^2^^,^^7^ Despite, elbow arthroscopy is generally considered a safe procedure with low complication rates.^8^

To our knowledge, with the present study, we lay out for the first time that a peripheral nerve block as part of arthroscopic arthrolysis for elbow stiffness, combined with a postoperative nerve block catheter, has a positive effect on regaining ROM in the affected elbow. Supporting these findings, we found a significant difference comparing the delta values of ROM arc for extension/flexion before surgery and after 6 months between both groups (*P* = .0199). Regarding MCID, the results demonstrate that group 1 clinically significantly benefited from the intervention after 6 months, while group 2 showed in contrast limited clinically significant improvement. This may be attributed to differences in preoperative ROMs for extension/flexion and pronation/supination. However, although group 1 had poorer preoperative ROM for extension/flexion, these patients matched group 2 in postoperative ROM and showed a greater overall improvement in ROM (Ex/Flex) 6 months postoperatively (see Δ-values, Table II). This suggests that the intervention was clinically more effective for group 1. Further indicating that effects are likely attributed to perioperative peripheral nerve blocks is the fact that the most drastic improvements in ROM were seen in the early postoperative phase. In particular, the large improvement of ROM Ex/Flex (Δ 17.78°) after 6 weeks in group 1, compared to group 2 (Δ 6.79°). These may be attributed to enhanced mobilization in early postoperative phase, resulting from more effective pain management through peripheral nerve block (Table II). Therefore, we conclude a possible significant influence of regional nerve block on postoperative outcome for this patient group. However, further research is needed to support and verify these findings. Studies evaluating postoperative pain after peripheral nerve block using standardized pain scores would be valuable, investigating the pain relief achieved through the nerve block catheter and its subsequent effect on mobilization in elbow surgery. Thereby, peripheral nerve block and catheter in addition to general anesthesia for arthroscopic arthrolysis in stiff elbows could potentially be established to sustainably improve postoperative outcome regarding elbow motion as well as long-term patient satisfaction.

### Limitations

This study has some limitations. We observed that patients in group 2 began with a higher average baseline ROM (ROM extension/flexion, *P* = .0348; ROM pronation/supination, *P* = .1453) in comparison to patients in group 1. However, as no significant differences were observed in the remaining preoperative characteristics, as shown in Table I, we can reasonably assume that the groups were comparable. This led us to hypothesize that the potential for enhancing elbow joint mobility in group 2 might have been relatively lower than in group 1. Consequently, the presence of a secondary bias cannot be entirely dismissed. We postulate that this observation could be linked to a correlation wherein patients with restricted preoperative mobility were more likely to have received a peripheral nerve block, potentially contributing to the described scenario. Although it is important to note that patients in group 1, despite initiating with preoperative values featuring an average ROM approximately 20° less in the ROM arc for extension/flexion compared to group 2, demonstrated comparable ROM after 6 months, reaching approximately 125°. Also, we acknowledge the relatively short follow-up duration of 6 months, which may limit the evaluation of potential worsening of initially good elbow function in long-term follow-up. Furthermore, dosage of anesthesia depended on individual patient conditions such as height and weight of the patient and the attending anesthesiologist. It is also important to note that the placement of regional anesthesia was performed by the same consultant anesthesiologist, with various attending physicians involved, which may have added variability in the quality of the nerve blocks. Also, surgical procedures were performed by 2 senior elbow surgeons. However, this variability reflects the standard conditions of clinical practice. Hence, we believe that this aspect can even be assumed as an advantage of the study, as we were still able to demonstrate significant differences between both groups. Moreover, the sample size of this study was restricted, so an effect of peripheral nerve block in context of arthroscopic arthrolysis of the stiff elbow on postoperative outcomes in larger samples is possible, although the clinical significance might be questioned. Therefore, our results must be confirmed in larger cohorts.

## Conclusion

Additional peripheral nerve block, coupled with a postoperative nerve block catheter in arthroscopic arthrolysis in cases of elbow stiffness, may be an opportunity for improving postoperative outcomes by achieving better functional ROM, perhaps through optimized postoperative pain management.

## Disclaimers:

Funding: No funding was disclosed by the authors.

Conflicts of interest: The authors declare that they have no known competing financial interests or personal relationships that could have appeared to influence the work reported in this article.

## References

[1] Akhtar A., Hughes B., Watts A.C. (2021). The post-traumatic stiff elbow: a review. J. Clin. Orthop. Trauma.

[2] Desai M.J., Mithani S.K., Lodha S.J., Richard M.J., Leversedge F.J., Ruch D.S. (2016). Major peripheral nerve injuries after elbow arthroscopy. Arthroscopy.

[3] Fredrickson M.J., Krishnan S., Chen C.Y. (2010). Postoperative analgesia for shoulder surgery: a critical appraisal and review of current techniques. Anaesthesia.

[4] Halvorson R.T., Lalchandani G.R., Cherches M.F., Petit L.M., Lattanza L., Lee N.H. (2023). Interobserver and intraobserver reliability of classification systems for radiographic complications after radial head arthroplasty. J. Hand Surg. Am.

[5] Haque R., Baruah R.K., Bari A., Sawah A. (2021). Is suprascapular nerve block better than Intra-articular corticosteroid injection for the treatment of adhesive capsulitis of the shoulder? A randomized controlled study. Ortop Traumatol Rehabil.

[6] Hastings H 2nd, Graham T.J. (1994). The classification and treatment of heterotopic ossification about the elbow and forearm. Hand Clin.

[7] Hilgersom N.F.J., van Deurzen D.F.P., Gerritsma C.L.E., van der Heide H.J.L., Malessy M.J.A., Eygendaal D. (2018). Nerve injuries do occur in elbow arthroscopy. Knee Surg Sports Traumatol Arthrosc.

[8] Intravia J., Acevedo D.C., Chung W.-L.J., Mirzayan R. (2020). Complications of elbow arthroscopy in a community-based practice. Arthroscopy.

[9] Itoi E., Arce G., Bain G.I., Diercks R.L., Guttmann D., Imhoff A.B. (2016). Shoulder stiffness: current concepts and concerns. Arthroscopy.

[10] Kalthoff A., Sanda M., Tate P., Evanson K., Pederson J.M., Paranjape G.S. (2022). Peripheral nerve blocks outperform general anesthesia for pain control in arthroscopic rotator cuff repair: a systematic review and meta-analysis. Arthroscopy.

[11] Kamat A.S., Jay S.M., Benoiton L.A., Correia J.A., Woon K. (2014). Comparative outcomes of ulnar nerve transposition versus neurolysis in patients with entrapment neuropathy at the cubital tunnel: a 20-year analysis. Acta Neurochir.

[12] Kim H., Kim H.-J., Lee E.-S., Lee S., Park J.H., Kim H. (2021). Postoperative pain control after arthroscopic rotator cuff repair: arthroscopy-guided continuous suprascapular nerve block versus ultrasound-guided continuous interscalene block. Arthroscopy.

[13] Leschinger T., Müller L.P., Hackl M., Wegmann K. (2016). Techniken der Arthrolyse am Ellenbogen. Obere Extrem.

[14] Morrey B.F., Askew L.J., Chao E.Y. (1981). A biomechanical study of normal functional ebow mo-tion. J Bone Jt Surg Am.

[15] Myden C., Hildebrand K. (2011). Elbow joint contracture after traumatic injury. J Shoulder Elbow Surg.

[16] Neviaser A.S., Neviaser R.J. (2011). Adhesive capsulitis of the shoulder. J Am Acad Orthop Surg.

[17] Okur S.C., Ozyemisci-Taskiran O., Pekindogan Y., Mert M., Caglar N.S. (2017). Ultrasound-guided block of the suprascapular nerve in breast cancer survivors with limited shoulder Mo-tion - case series. Pain Physician.

[18] Sanchez-Sotelo J. (2021). Arthroscopic management of elbow stiffness. J Exp Orthop.

[19] Sardelli M., Tashjian R.Z., MacWilliams B.A. (2011). Functional elbow range of motion for contemporary tasks. J. Bone Joint Surg. Am.

[20] Steinmann S.P., Adams J.E. (2020). Editorial commentary: elbow arthroscopy is a safe procedure. Sure. Arthroscopy.

[21] Sun Z., Cui H., Liang J., Li J., Wang X., Fan C. (2019). Determining the effective timing of an open arthrolysis for post-traumatic elbow stiffness: a retrospective cohort study. BMC Musculoskelet. Disord.

[22] Sun Z., Li J., Luo G., Wang F., Hu Y., Fan C. (2021). What constitutes a clinically important change in Mayo Elbow Performance Index and range of movement after open elbow arthrolysis?. Bone Joint Lett J.

[23] Warner J.J., Allen A., Marks P.H., Wong P. (1996). Arthroscopic release for chronic, refractory adhesive capsulitis of the shoulder. J. Bone Joint Surg. Am.

