# Supplementary material for: A comparative analysis of short-term results in range of motion following arthroscopic arthrolysis with vs. without peripheral nerve block in cases of elbow stiffness
Source: JSES Int. 2024 Nov 16;9(2):568–73. doi: 10.1016/j.jseint.2024.10.009 (PMC11962604; doi:10.1016/j.jseint.2024.10.009)
Supplement: Supplementary Table S1 [file mmc1.docx]

**Supplementary material:**

**Supplemental Table 1:** ROM and DASH compared to preoperative status. Comparison of both groups.

|  | Pre OP | 6 weeks | p-value | 3 months | p-value | 6 months | p-value |
| --- | --- | --- | --- | --- | --- | --- | --- |
| **Group 1** |  |  |  |  |  |  |  |
| ROM Ex/Flex | 95° (±27.17) | 112.8° (±18.4°) | 0.0335 | 118.9° (±15.6°) | 0.0008 | 124.4° (±12.7°) | 0.000012 |
| ROM Pro/ Sup | 150° (±29.1) | 161.1° (±18.4°) | 0.0768 | 161.7° (±19.2°) | 0.0634 | 170.6° (±13°) | 0.0013 |
| DASH | 37.47 (±20.33) | 27.44 (±20.22) | 0.9952 | 16.24 (±16.22) | 0.1175 | 11.55 (±7.71) | 0.0159 |
| **Group 2** |  |  |  |  |  |  |  |
| ROM Ex/Flex | 113.9° (±15.2) | 120.7° (±9.9°) | 0.9586 | 126.8° (±9.9°) | 0.4481 | 125.4° (±12.5°) | 0.6016 |
| ROM  Pro/ Sup | 161.1° (±40.9) | 173.9° (±9.6°) | 0.071 | 174.6° (±11.8°) | 0.057 | 169.3° (±22.3°) | 0.2461 |
| DASH | 36.97 (±26.09) | 34.99 (±26.02) | 0.9999 | 28.31 (±28.77) | 0.9999 | 20.39 (±21.85) | 0.749 |
